# A Practical Format to Organize Cancer Constellations Using Innate Immune System Biomarkers: Implications for Early Diagnosis and Prognostication

**DOI:** 10.3390/ijtm4040050

**Published:** 2024-12-06

**Authors:** Martin Tobi, Harvinder Talwar, Noreen F. Rossi, Warren Lockette, Benita McVicker

**Affiliations:** 1 Department of Research and Development, Detroit VAMC, 4646 John R Street, Detroit, MI 48601, USA; 2 Department of Research and Development, Wayne State University School of Medicine, Detroit VAMC, 4747 John R Street, Detroit, MI 48602, USA; 3 Division of Nephrology, Department of Internal Medicine, Research and Development, Wayne State University School of Medicine, Detroit VAMC, 4646 John R Street, Detroit, MI 48601, USA; 4 Division of Endocrinology, Department of Internal Medicine, Research and Development, Wayne State University School of Medicine, Detroit VAMC, 4646 John R Street, Detroit, MI 48601, USA; 5 Research Service, VA Nebraska-Western Iowa Health Care System, University of Nebraska Medical Center, Omaha, NE 68105, USA

**Keywords:** cancer constellations, p87, Adnab-9, FERAD ratio, absolute neutrophil/lymphocyte ratio, PD-L1 treatment outcomes

## Abstract

Cancer discovery is directed at the identification of a specific cancer type which allows for specific therapeutic interventions. Background/Objectives: Recently, similar immune checkpoint therapeutics have been applied with success across several cancer types, opening the field for other immune disruptive interventions that have practical applications. Methods: We have discovered an innate immune system (InImS) biomarker that allows for the characterization of allied cancer subtypes and outliers that might aid with diagnosis, treatment, and prognostication. Results: These InImS biomarkers are related to PD-L1 treatment outcomes and can be potentially manipulated by dietary means. Conclusions: The FERAD (ferritin–fecal p87) and absolute neutrophil/lymphocyte (aNLR) ratios are two such InImS biomarkers and we show herein, that they allow for the discovery of diagnosis and prognostication patterns, as demonstrated by this study.

## 1. Introduction

The teleological fragmentation of the medical efforts in cancer discovery and treatment has been tempered by the understanding that the human body has the capacity to recognize a tumorigenic process and release the key immunogenic factors blocked by neoplastic subterfuge [1]. Once these factors are released, their activation allows for a unique and effective counterattack. Our emergent understanding of InImSs was a silver lining ushered in by the disastrous COVID-19 pandemic cloud, where we showed that both the FERAD and absolute neutrophil/lymphocyte ratios [2] were biomarkers for the susceptibility and severity of COVID-19 in patients [3]. They also happen to be prognostic for a number of cancer types [4,5]. Fortuitously, we had assembled a relatively large database of Veterans at increased risk for colorectal cancer (CRC) from a prospective study in which the patients were followed for almost 3 decades. As part of this study, stool and effluent samples (colonoscopically collected colonic washings) were collected and the patients were followed for a period of time, chronicling any new cancer diagnoses and allowing for the establishment of a window to compare the baseline stance of the InImS using the abovementioned ratios and its relationship and prognosis vis a vis the encountered cancer [6]. We therefore undertook to characterize a number of common malignancies to try to establish cancer behavior patterns and correlate these with the overall survival outcomes for patients using immune checkpoint blockers from the data available in the online literature. It was also important to examine whether the ability to define cancers with shared survival trajectories (constellations) also extends into the realm of distant tumor metastasis, and hence, prognosis. We obtained historic ferritin blood levels and stool p87 samples for the following purposes: to derive the patients’ FERAD ratios (ferritin–p87) at the time of enrollment to obtain an exact snapshot of their InIMSs at the earliest time possible; to be able to correlate these ratios with the contraction of future cancer as derived from the database updates. We thus derived cancer constellation formations, which became a major aim of this novel study. This allowed us to make comparisons with the published survival data, gleaned from the PD-L1 interventional trials available on the internet [7]. On the downside, when conventional therapy fails and it prognostically appears that the cancer has overwhelmed the InImS in cases of distant metastasis to the liver, a common harbinger of death. The manipulation of the InImS, theoretically, may be beneficial and thus, may have clinical applicability.

## 2. Materials and Methods

### 2.1. Database

We obtained a database of patients at high risk of colorectal neoplasia and prospectively noted those who contracted cancer on follow up. We contrasted the historic, premorbid biomarker response from the time of enrolment, primarily using the FERAD ratio. This was obtained by dividing the ferritin blood concentration by the denominator of the fecal shedding of a Paneth cell marker, p87. We also compared the FERAD response to the absolute neutrophil/lymphocyte ratio (aNLR), a known prognostic biomarker, and the available PD-L1 response for each group of cancers. The latter was obtained through an online search.

### 2.2. Sample Collection

Briefly, stool and other bodily fluids were obtained after a consent form was read by the participant who then signed the form. The inclusion criteria were a willingness to participate in a long-term observation study and to undergo a colonoscopy which was ordered by a primary-care provider (PCP). There was a phase-2 part of this study where 10% of the enrollees were asked to permit regional biopsies of their colons to be taken via a forceps biopsy for immunohistochemistry and non-fixed tissue extraction. The timing of the second colonoscopy was left to the shared discretion of the PCP and the patient. Needless to say, the times for voluntary procedures differed widely but almost 50% of the enrollees complied.

### 2.3. Exclusion Criteria

These were based on the state of health of a patient that may have excluded the possibility of a colonoscopy, including the inability to write the date on the stool cards, and the inability to be in compliance with the standards of this study. Colonic washings were also collected at the time of the endoscopy from the pool that usually collects in the rectum.

### 2.4. Storage and Other Samples

Saliva and urine samples were also collected and all the samples, where appropriate, were stored at −70 °C. We were able to show in preliminary reports that the stored samples gave identical results after 10 years of storage and that the p87 protein was not affected after being held at room temperature for 6 days. This was important as most participants mailed in their stool cards and the outer limit of mail delivery in our locale was 5 days.

### 2.5. ELISA (Enzyme Linked Immunosorbent Assay) and Antibodies

The ELISA testing was performed in accordance with the manufacturers’ directions and involved the determination of the protein content of stool diluted in phosphate-buffered saline which allowed us to plate 5 μg of protein per well of a 96-well Nunc microtiter plate Catalogue GS-120030 from Oldsmar, FL, USA. The plates were incubated overnight at 4 °C and the Adnab-9 antibody that recognizes the p87 antigen was placed on half of the plate which had been blocked with a 5% bovine serum albumin solution. All the washes between the primary and secondary antibodies were performed with PBS and 5% Tween-20 (MilliporeSigma, Catalogue 93773, Burlington, MA, USA). The reagents were supplied by Vector Laboratories (Catalogue SA-5014-1, Newark CA, USA) for immunohistochemistry by the ABC technique using their instructions, The final step after the sandwich ELISA had been achieve was to add 40 μL of p-nitro-phenyl phosphate (MilliporeSigma, Catalogue N7653, Burlington, MA, USA) to derive a yellow color that was read on a Thermo-Fisher cyto-spectrophotometer at 405 nm. The negative control antibody, UPC10 (MilliporeSigma, Catalogue M9144, Burlington, MA, USA), was an indifferent antibody with the same isotype as Adnab-9 (IgG2a) and was applied to half of the plate which was subtracted from the reading from the other half of the plate that had been treated with Adnab-9. The results were expressed as the optical density (OD) minus the background.

### 2.6. Western Blotting

The same reagents were used to run Western blots on Bio-Rad electrophoresis equipment and transfer the blotters using 10% nitrocellulose gels, incorporating the molecular weight markers (BIORAD 1620115, Hercules, CA, USA) and PVDF membranes (BIORAD 1620184, Hercules, CA, USA). The FERAD InImS marker was also combined occasionally with the product of the FERAD ratio and NLR or by division, to explore for additional biomarker advances.

### 2.7. Institutional Review and Statistical Analysis

The Institutional Review Boards at Wayne State University and other participating medical centers reviewed and approved the above protocols. The results were analyzed using an Instat Inc statistical package. The ordinal data conforming to normality were analyzed by a two-tailed Student’s *t*-test and the non-ordinal date were analyzed by a Chi-square test. *p*-values < 0.05 were regarded as positive. Where samples were limited, we occasionally used a 1-tailed *t*-test if the results were congruous with similarly measured trends.

## 3. Results

**Figure 1.**
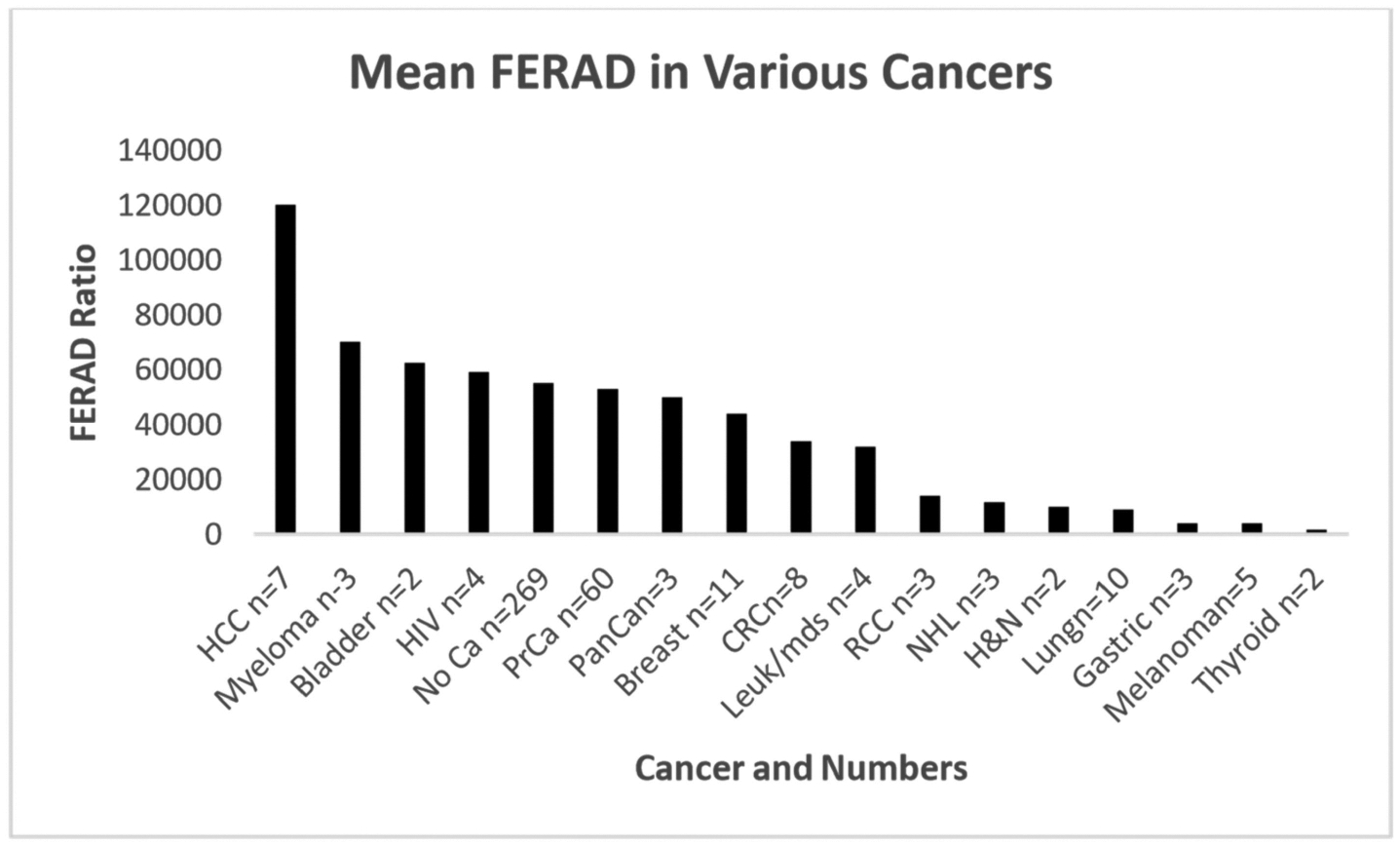
Comparative FERAD ratios associated with cancer types.

We found that certain groups of cancers historically had very low FERAD ratios while others were high as shown in the bar diagram below (Figure 1).

This figure shows the past response of the InImS from the time of the initial stool sampling before the cancer diagnosis. The highest preemptive ratios are seen with hepatocellular cancers (HCC), melanoma, and bladder cancer. For comparison, HIV patients (treated) and patients without cancer follow the first two cancer group.

Following the controls are prostatic cancer, pancreatic cancer, breast cancer, CRC, and blood dyscrasias. The last group with decidedly low FERAD ratios (<10,000) are renal cell cancer, non-Hodgkin’s lymphoma, head and neck cancer, lung cancer, gastric cancer, and thyroid cancer. The best cancers characterized thus far with respect to FERAD ratios have been gastric cancers with and without Helicobacter pylori [5]. We correlated the FERAD scores with the observed survival times from the database to check for correlation as described below.

**Figure 2.**
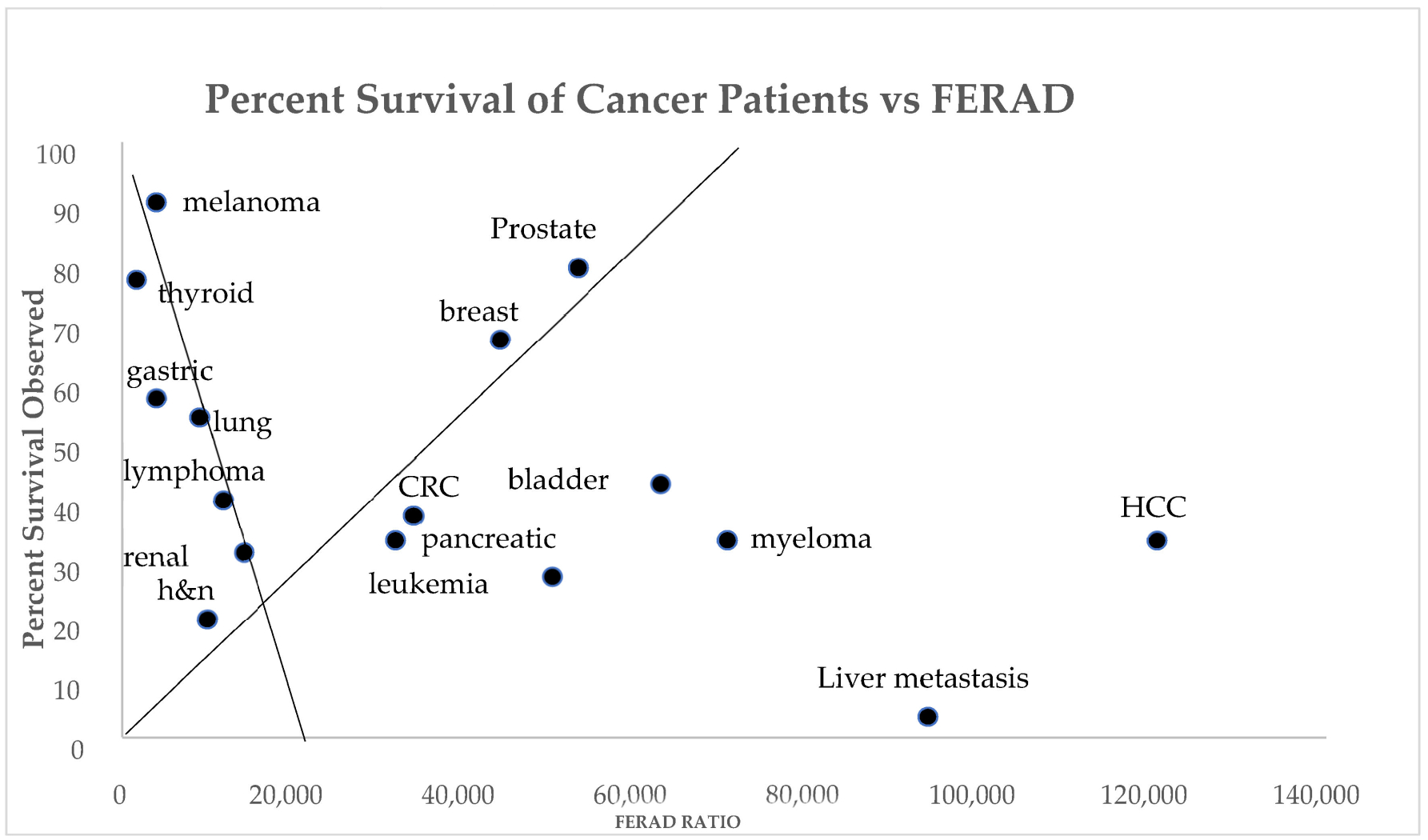
A scattergram showing 2 constellations of cancer correlations with Liver Cancer Outliers. Abbreviations: h and n—head and neck cancers; HCC—hepatocellular cancer.

Using the FERAD ratios for lung cancer, gastric cancer, non-Hodgkin’s lymphoma, melanoma, head and neck cancer, renal cancer, and thyroid cancer, there is a significant inverse correlation between the survival rate and FERAD ratio (r = −0.8; *p* < 0.025) as seen in Figure 2. For prostate cancer, breast cancer, colorectal cancer (CRC), and leukemia/MDS, there is a direct correlation between the survival rate and FERAD ratio (r = 0.99; *p* < 0.002).

We obtained the 5-year percent survival rates and plotted them against the available mean FERAD ratios for each cancer. Interestingly, we found that there were two main cancer continua, those with a low FERAD ratio (<2000) driven by melanoma and thyroid cancer had a poorer survival rate while a lower FERAD ratio driven by prostate and breast cancer (5000–7000) had a better survival. The mid-region (30–40% survival rates) include renal cancer, CRC, pancreatic cancer, leukemia, myeloma, and HCC. Compared to the rest, liver metastasis survival is the most dismal. There is a desperate need to address this sad reality and to exploit this and other markers in the crusade moonshot current mindset.

**Figure 3.**
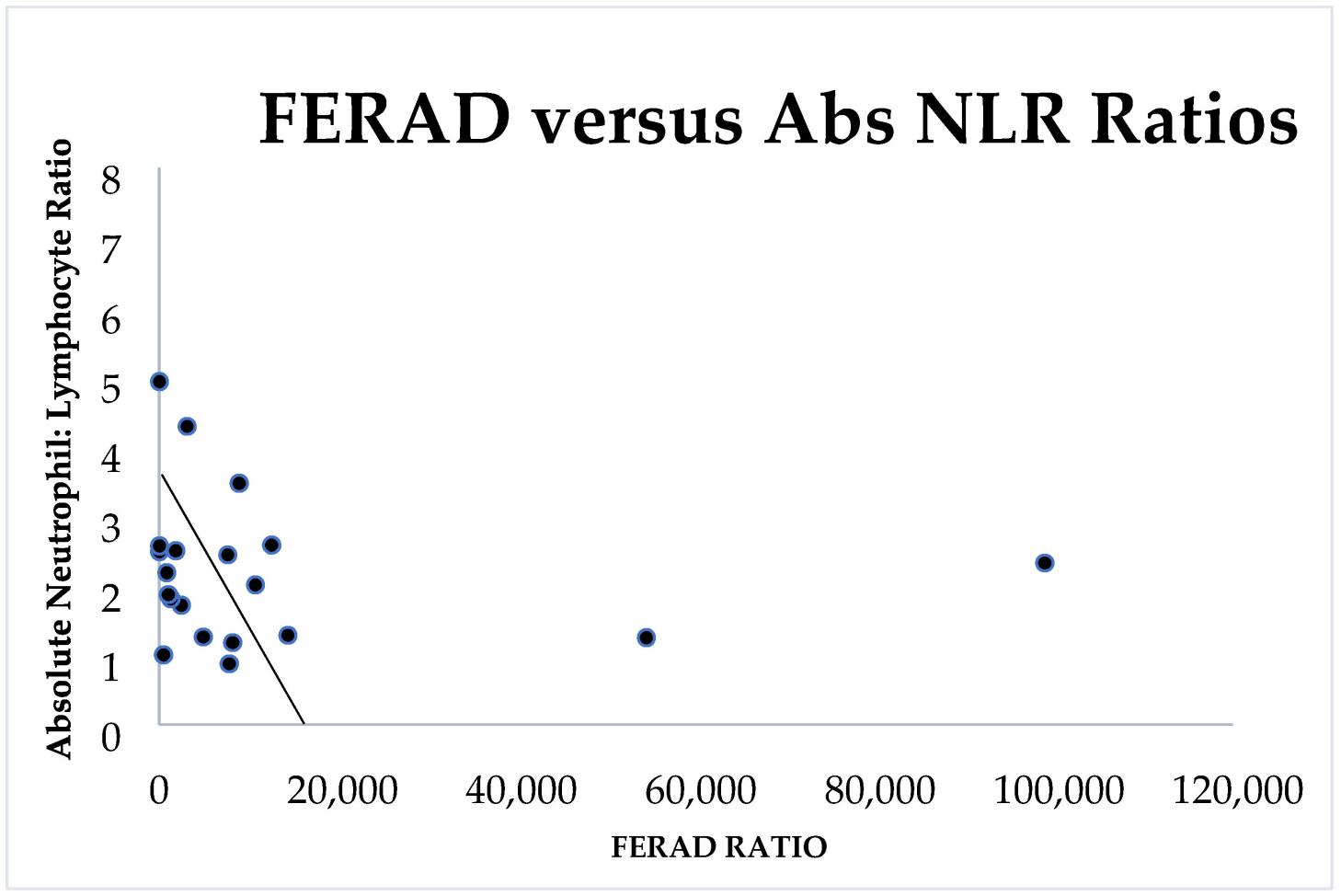
A scattergram showing an indirect correlation between FERAD and absolute NLR.

We therefore plotted the FERAD ratios against another established prognostic marker, the absolute neutrophil/absolute lymphocyte ratio (NLR) as previously reported [3] in the same patients. The results are depicted in Figure 3.

**Figure 4.**
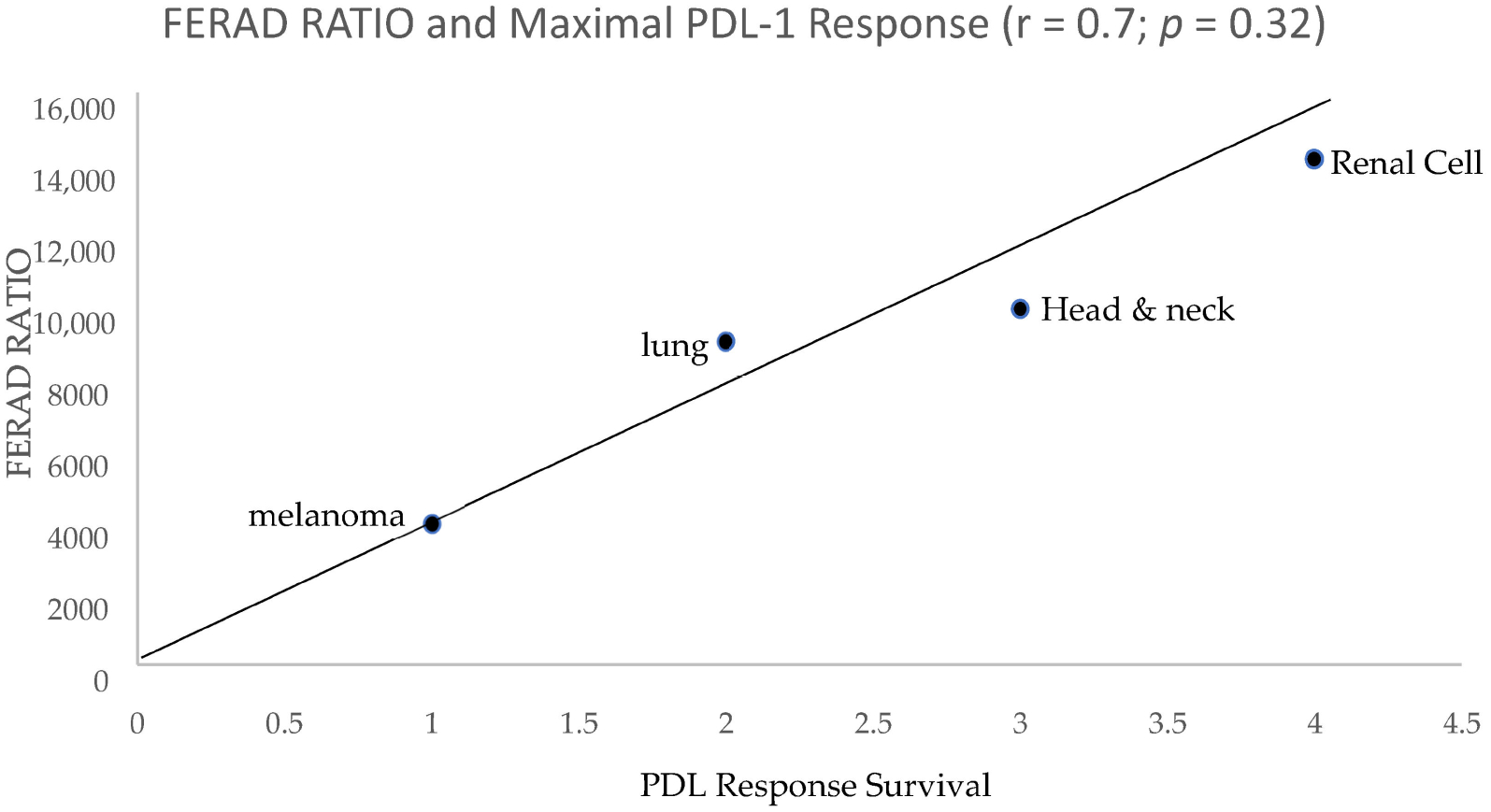
A scattergram showing selected PDL response versus FERAD ratio.

There is an inverse relationship between the NLR and PDL-1 response rates (r = −0.8; *p* < 0.04) as seen in Figure 3. The two markers bear a correlation, and both are derived from the constituents of an InImS but their predictions are diametrically opposite and may be interpreted as FERAD taking the “long view” while NLR, takes the “shorter”. The two outliers are of interest since the prognosis is better with a low NLR (usually <3) and is likely better where the FERAD ratio is elevated, but there are exceptions (liver metastasis and HCC) as can be seen in Figure 1 (HCC) and Figure 2 (both). Interventions for highly lethal tumors are now in vogue with cancer immune checkpoint (CIC) inhibitors leading the charge. In case of point, an observed mild positive trend for FERAD PDL-1 response rates may bode well for melanoma, lung cancers, head and neck cancers, and renal cancers (Figure 4).

From this graph, we learn that there appears to be a positive relationship between the bedrock FERAD and current beneficial PDL-1 response, led in this instance by head and neck cancer and renal cell cancers. It is important to dissect the basic congeners of the FERAD ratio (highly specific stool (by Western blotting)) and colonic effluent p87 to see if they are likely acting in concert to yield prognostic data that may direct future immune interventions.

**Figure 5.**
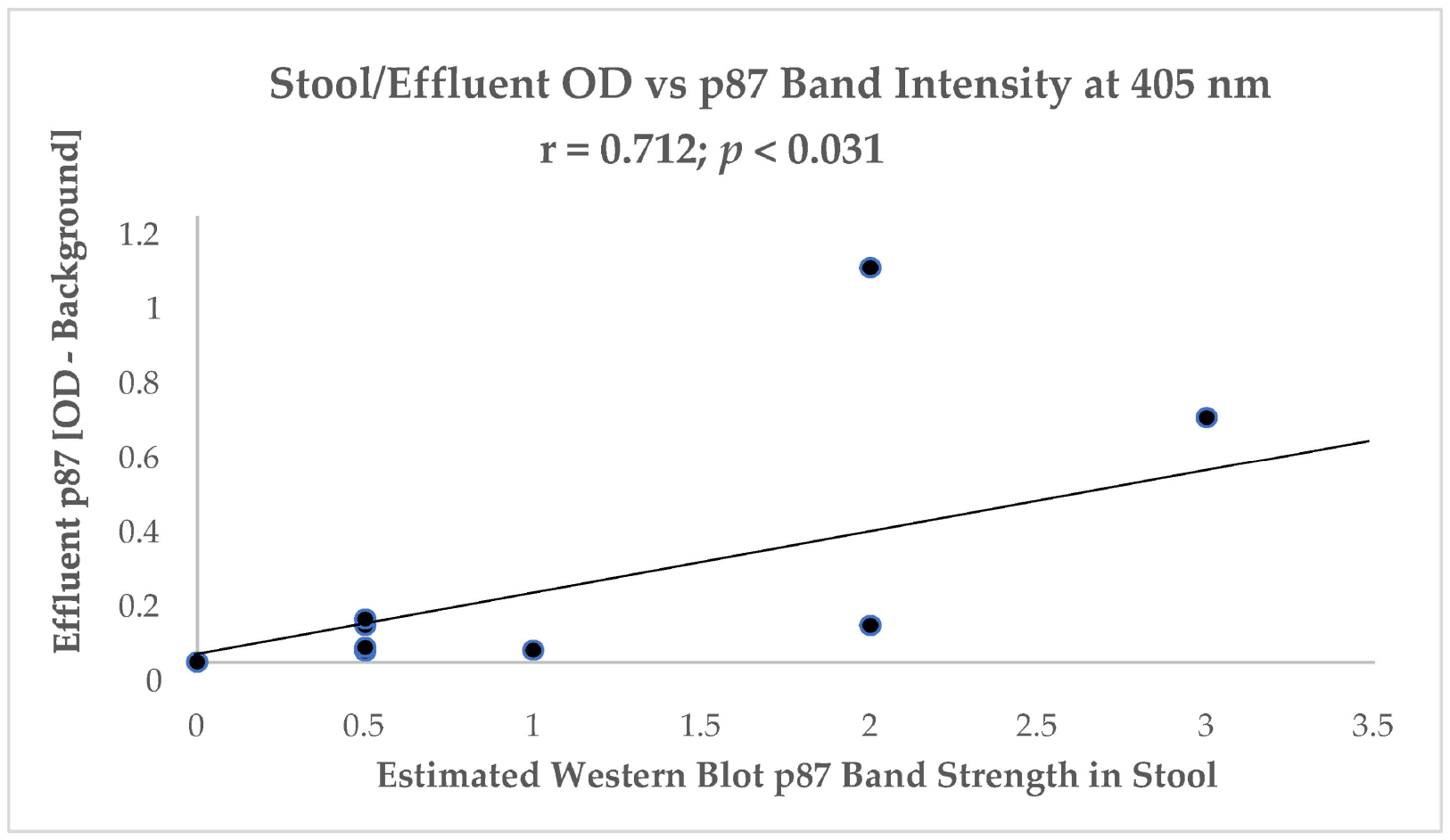
A correlation between stool and colonic effluent.

Figure 5 shows a trend toward a positive correlation between stool and colonic effluent ELISA correlated with the estimated intensity of the specific bands on the Adnab-9 Western blots.

These data suggest that the positive relationship between the different assays for the p87 and other protein bands, as detected by Adnab-9 binding, is specific and either one or both should be considered as likely prognostic markers.

While we have shown an inverse relationship between the NLR and FERAD ratio (Figure 3), we have not proven an existing relationship of an independent NLR-related cancer constellation. In order to achieve this, we looked at an extensive meta-analysis of the NLR [8] and were able to obtain NLR-related Hazard Ratios.

**Figure 6.**
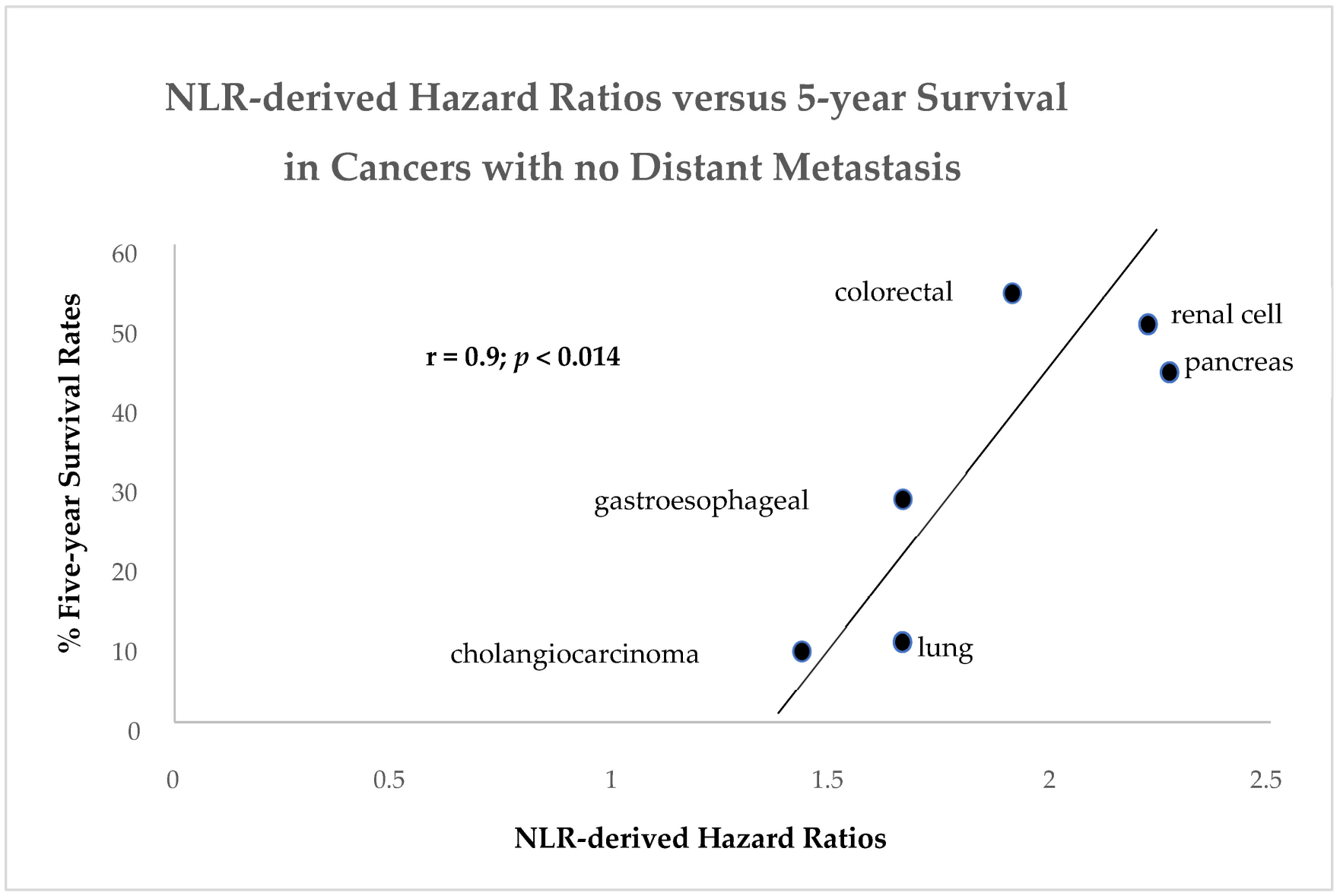
A linear regression plot of NLR-related Hazard Ratios versus 5-Year cancer survival rates.

Using the ratios for common cancers and the ACS and other online database reports on 5-year survival rates (https://www.cancer.org/cancer/types/kidney-cancer/detection-diagnosis-staging/survival-rates.html; and https://ourworldindata.org/grapher/five-year-survival-rates-by-cancer-type, accessed on 18 August 2024), we were able to plot a linear regression curve showing a significant direct relationship as seen in Figure 6.

As the ratio increases, there is a relative increase in the survival rate. The stages of each disease were not uniform in this opportunistic plot but overall, the results were similar to the FERAD ratio constellation. The plot could also be interpreted as highly selective, favoring any NLR < 3 for certain cancers with the appearance of a better survival rate. Clearly, we needed to expand our investigation by broadening the FERAD-NLR horizon.

We sought to expand the FERAD ratios and NLRs into a cooperative utility and identified the patients with cancer who had both FERAD ratio and NLR results. We then either multiplied the ratios and achieved a bimodal score (BM) or divided the FERAD ratio by the NLR to yield another permutation, the ratio composite (RC). While not successful in achieving a complete agreement with the above expanded prognostic scores, we were rewarded with several statistically positive outcomes when combing the ratios (please see Table 1).

The cancers were compared to the controls and the groups that were statistically different from the controls were colorectal, bladder, melanoma, and to a certain extent, breast.

**Figure 7.**
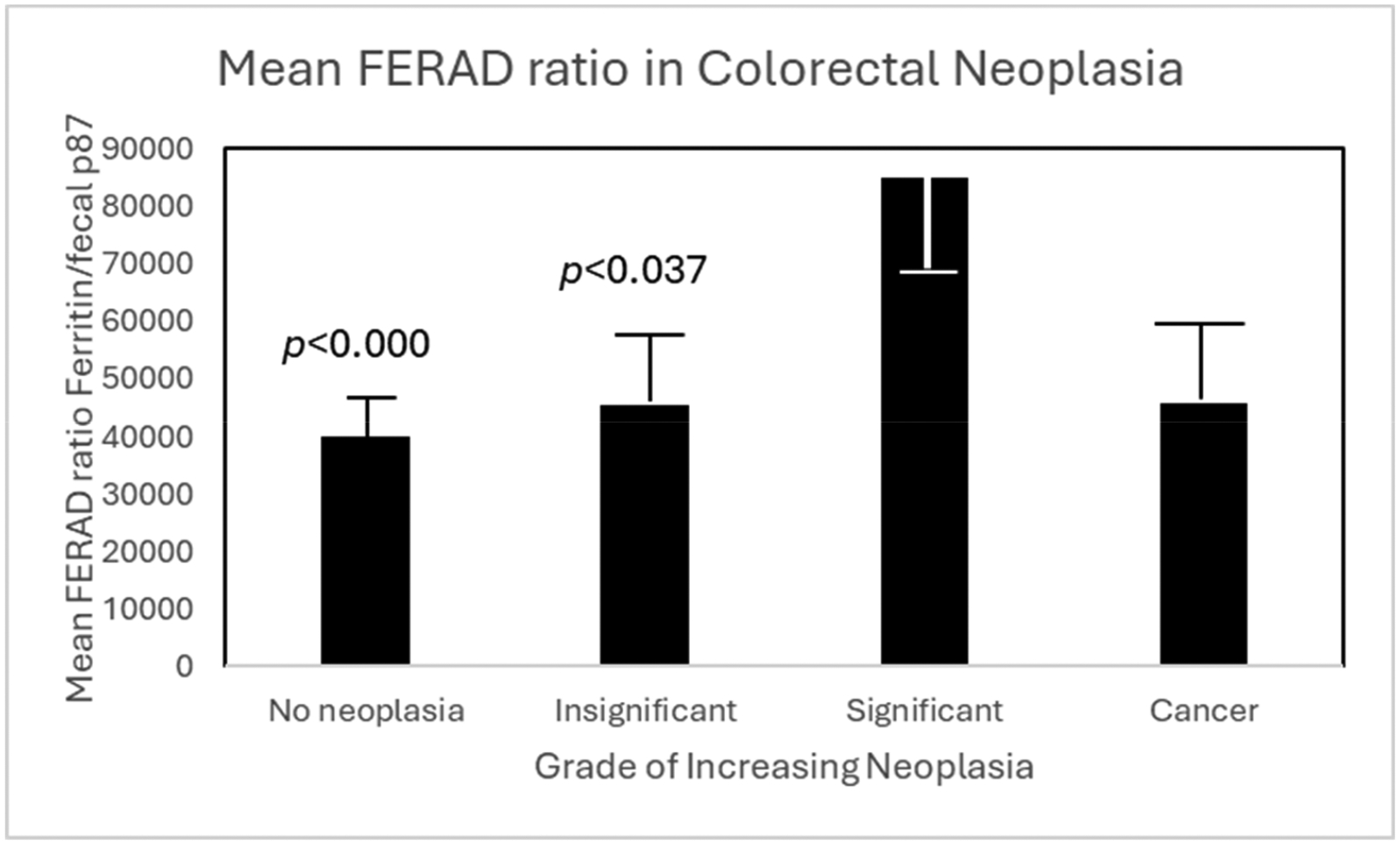
A bar diagram showing the FERAD scores in various stages of malignancy.

While cancer data are important to the understanding of the workings of InImSs, the lead-up to cancer in the guise of premalignancy and its early detection are more practical in this study which could lead to early intervention. Our biomarkers of choice were therefore investigated after reviewing the current panoply of available biomarkers with extended relevance to the immune system [9]. In 2015, publications on this topic surged to 2637, but since then there was a decline to 2029 in 2023 (https://pubmed.ncbi.nlm.nih.gov/?term=Premalignancy, accessed on 15 May 2023) when AI entered the fray. Currently, oral premalignancy dominates, but novel biomarker detection using transcriptomic searches has opened the playing field [10]. But their proof-of-concept publication was mainly centered on familial adenomatous polyposis (FAP) syndrome derivatives. Nevertheless, we will draw on our resources to present the intercept of the well-characterized adenoma carcinoma sequence with premalignancy and the InImS as shown in Figure 7.

The sequence begins with no observed polyps or hyperplastic polyps of <1 cm in size and left-sided followed by insignificant adenomas of <1 cm in size without severe dysplasia (HGD) upon histologic examination; significant adenomas are those of >1 cm in size, more than two adenomas, or HGD. The colorectal cancer cases are inserted for comparison. The FERAD ratio is significantly lower in the premalignant states compared to states of significant disease. These data were derived from our recently published NIPCON (Non-Invasive Prediction of Colorectal Neoplasia) trial [6]. Please see Table 2 for patient details and additional statistical analysis.

Since the adenoma–carcinoma sequence groups bore no statistical significance aside from the expected age differences, we extended our attention to other premalignant states of the gastrointestinal tract (GIT).

**Figure 8.**
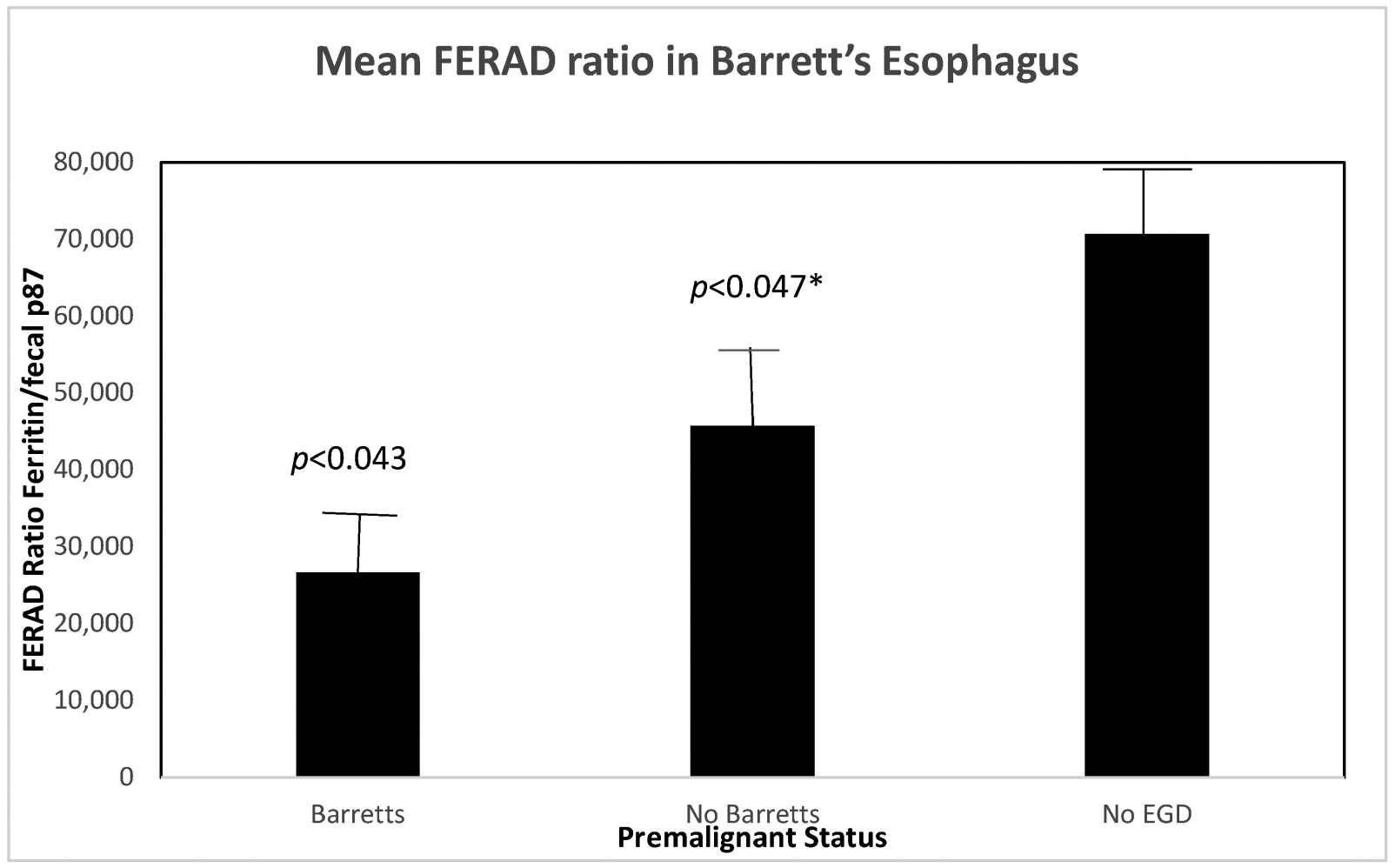
The lowest FERAD ratio levels are seen in patients with Barrett’s esophagus. The * designates the *p*-value as a one-tailed Student’s *t*-test.

Another undisputed example of premalignancy is that of Barrett’s esophagus (BE), in which we greatly detail this entity and promote the measures for regression currently In-Press [11]. We computed the FERAD ratio for 276 patients who did not undergo an esophagogastroduodenoscopy (EGD), with 85 who had BE and 72 who had no findings of BE on their EGD as depicted in Figure 8.

While the above results support the notion of reduced InImS surveillance for Barrett’s esophagus and thus, increase the risk of highly lethal esophageal cancer, we took the next step of descent through the GIT to try to apply the FERAD response to premalignant conditions in the stomach.

We have previously published gastric FERAD ratios, including the types of cancers [5] and further details, which are provided in Table 3 below in terms of gastric premalignant lesions.

In comparison, the patients with elevated serum gastrin concentrations of >180 pg/mL had a FERAD ratio of 66,511 ± 102,587, n = 6, and the patients with pancreatic cancer had a FERAD ratio of 35,229 ± 56,684, n = 7. The differences are not significant.

Since the cancer constellations of distant metastasis are not clearly defined, we studied CRC liver metastasis. We were able to muster 44 patients with liver metastases, where 12 patients had a questionable source for the primary neoplasm and 32 had well-characterized sources for the primary neoplasm.

**Figure 9.**
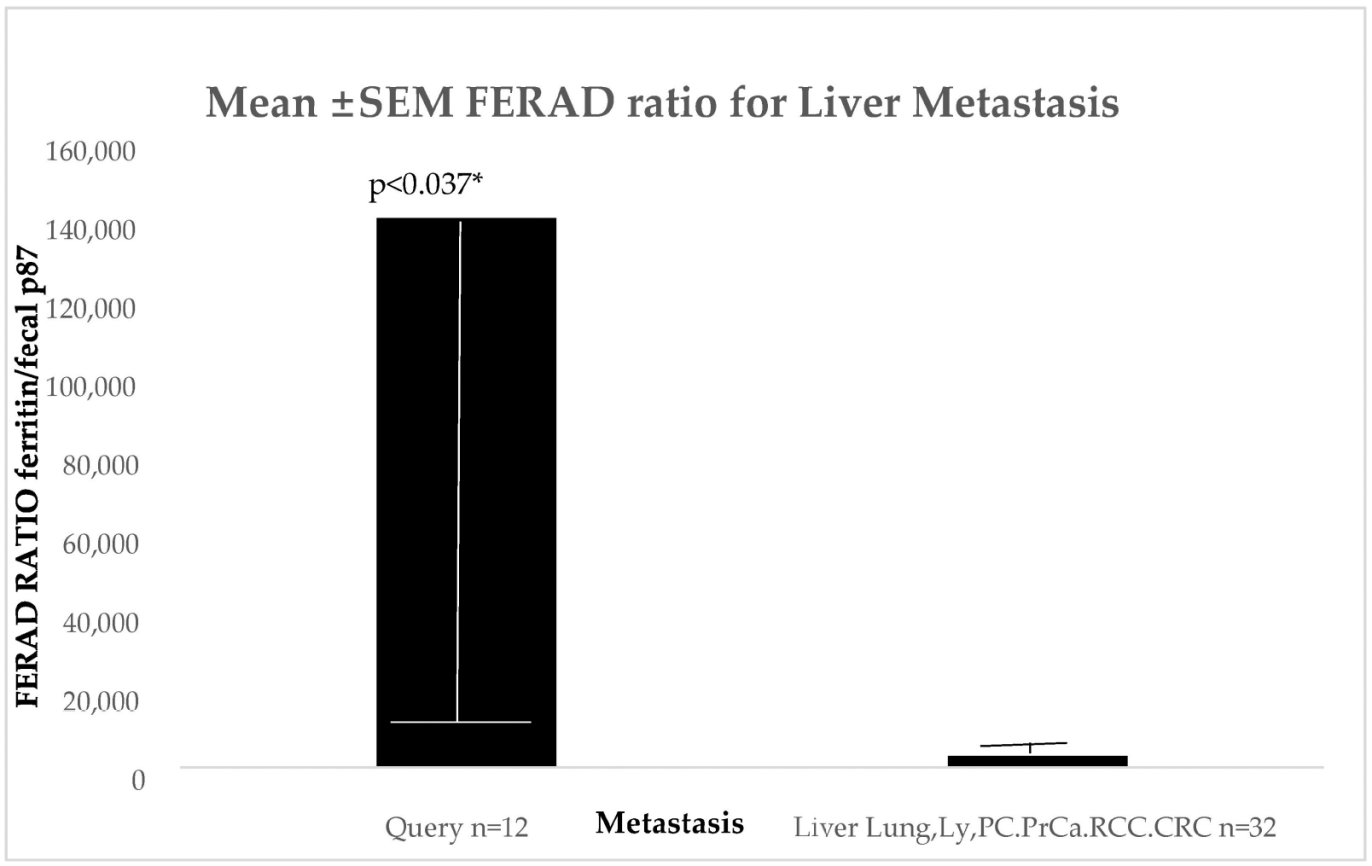
A bar diagram depicting the FERAD ratio value differences between patients with known and imprecisely known primaries. * *p* < 0.01.

Figure 9 shows the one-sided significance indicated by an Asterix.

Table 4 below provides the patient information germane to the background of the patients.

**Figure 10.**
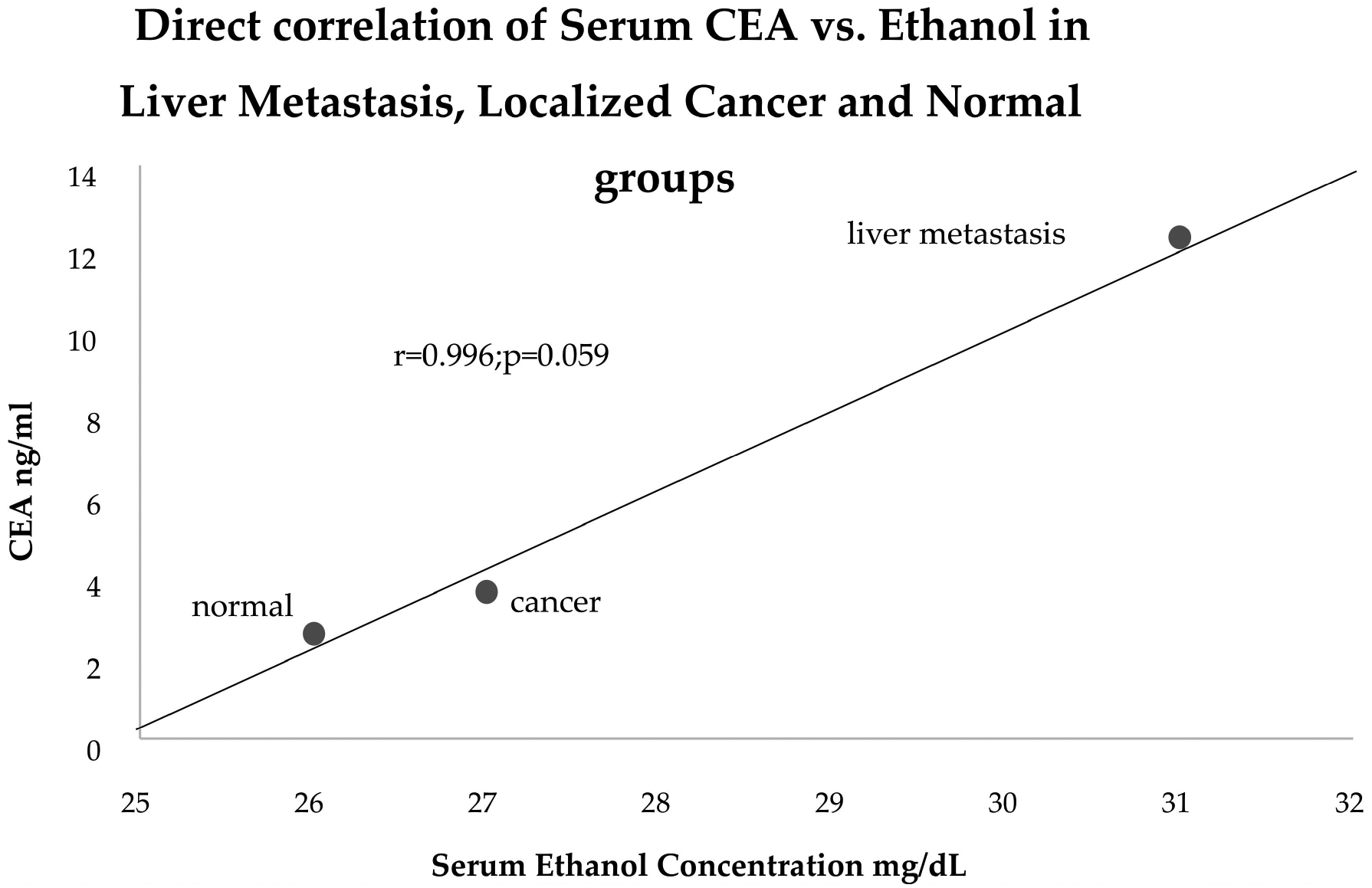
A linear correlation graph of the relationship between CEA and alcohol intake, which are both pro-metastatic.

Other than a significant difference in the observation time, which did not correlate with a prognosis, the two groups were otherwise identical aside from their close to significance FERAD ratio. There were, however, close correlations between the various groups of patients and alcohol content, which are shown in Figure 10.

There is a close relationship, generally speaking, between alcohol intake and CEA, which has long been recognized as a pro-metastatic promotor.

Since HCC presents an interesting group, we looked at the FERAD ratios in the patients with both cirrhosis and HCC. As can be seen in Table 5, the overlap group had the lowest FERAD ratio, which may explain the observed poor survival rates [12], despite their young age status of 48 years as seen in Table 1.

These results explain the relatively high FERAD ratio in the first two tables were in patients with cirrhosis who had been inadvertently included with the HCC patients. The low FERAD ratio in the overlap of the HCC and cirrhosis groups supports the above finding of a poor survival rate.

## 4. Discussion

Since p87 can be manipulated by dietary means [11] and possibly affect the FERAD ratio favorably, this could be incorporated into more effective chemotherapy regimens and result in better cancer treatment outcomes. Hepatocellular cancer (HCC) and CRC liver metastasis appear to be immunological outliers. This will need an additional unique approach such as TIGIT (a T cell immunoreceptor with immunoglobulin and ITIM domains), or intervention therapy [13]. This may result in the modulation of an immune response based on the FERAD ratio, NLR, and other considerations of mutational burden and genetic mutations in patients with selected cancers.

The forerunners of the FERAD ratio were based on the studies using the Adnab-9 monoclonal antibody to generate a gastric diagnostic prediction using stool concentrations for colorectal neoplasia [14], and prognostic immunohistochemistry labeling [5]. These publications drew a favorable, independent review of Adnab-9 applications as reported in the general medical literature [15] and others have independently confirmed the applicability of the predictive value of p87 as recognized by the monoclonal antibody, Adnab-9 [16]. It is on this solid basis that we have been able to expand our current observations. For example, as can be seen above, the relationship of renal cell cancer, head and neck/gastroesophageal cancers, and lung cancers were maintained, like those seen in Figure 4. This contrasted the PD-L1 survival rates with the FERAD ratios. In the direct relationship line, as plotted in Figure 2, CRC is followed by pancreatic cancer and then head and neck cancer, although lung cancer appeared on the inverse correlative line. In Figure 1, which compares FERAD ratios, pancreatic cancer precedes CRC, renal cancer, and head and neck cancer/lung cancer, and this anatomic relationship, while imperfect, tends to hold true.

Much of this work was completed in the VHA and at centers where AA are in the majority. The third leading cause of cancer-related deaths in Veterans is colorectal cancer, with 433,000 of the one million Veterans with CRC succumbing to the disease. The most common cause of CRC death [12,17] is the development of colorectal liver metastasis (CRLM).

The majority of deaths are due to colorectal liver metastasis (CRLM) as the liver is the foremost site of distant CRC spread [18]. Despite advancements in surgical interventions and chemotherapeutics, CRLM remains a leading healthcare concern, emphasizing the need to define the relevant mechanisms. Notably, alcohol consumption has been identified as an independent and significant risk factor for CRC metastatic disease [19,20]. This is why we chose to close Section 3 with Figure 10 which is an important concomitant of cancer and its spread.

As a biomarker of the InImS, the FERAD ratio is gaining significance amongst an ever-increasing repertoire which recently highlighted gastric cancer [21] and, as Figure 1 shows, the right-hand side of the graph shows that gastric cancer along with melanoma, lung cancers, and thyroid cancers have a barely noticeable InImS response. Figure 2 for the first time shows that there are constellations of cancer that follow a continuum for better prognosis, probably represented by better treatment strategies, but the bottom of the graph occupied by leukemias, hepatocellular cancer (HCC), liver metastasis, and pancreatic cancer, despite advances with the latter [22,23], still leave much to be desired in the failure to effectively treat these outliers, particularly HCC [24]. The absolute neutrophil/lymphocyte marker has its uses and we have shown above that it can join FERAD as a biomarker, despite their inverse relationship (Figures 3 and 6), but the cutoffs are not agreed upon and it is not yet ready for prime time.

## 5. Conclusions

The relationship between the FERAD ratio and PDL-1 response appears to be interesting but more data are needed to clinch the nature of the relationship. Using Western blotting to cement the relationship between the detectable p87 bands and colonic washings points into a solid partnership has led to successes, but still needs more development despite our success at detecting adenomas using the stool p87 biomarker [6]. Table 1 shows that permutations of the combinations of the FERAD ratio and NLR can be functional and expand the diagnostic vistas for breast, colorectal, bladder, and melanoma neoplasms. Figure 7 gives further support to the notion of the detection of premalignancy intervening in the adenoma cancer sequence and early diagnosis by occult blood studies combined with FERAD surveillance may represent a disruption of the field. Another early detection target may be the relationship of FERAD to “normal” and to Barrett’s esophagus. This is enticing as it may lead to identifying low FERAD ratios in patients for timely EGD surveillance and offers a chance to modulate the FERAD ratio via dietary means [11]. Likewise, we have shown that there is a decreasing FERAD continuum from premalignancy to gastric cancer as shown in Table 3. This is an important cancer worldwide and feasible early detection needs to be addressed [5]. Finally, we see that cancers of unknown primaries (CUPs) have a significantly greater FERAD score when compared to several lethal cancers as seen in Figure 9, but that does not suggest that CUPs are any less benign than its deadly counterparts. We could, likely, have an advanced warning a cancer that is lying and growing under the radar in an undisclosed location. It does appear from Table 4 that we may have additional time to intervene with CUP, but we should not tarry.

We present this hopefully comprehensive and daunting study, with relatively few precedents, as a beacon for those who would act on these findings as it is only with fellow scientists and willing minds that we can defeat the terrible scepter of cancer.

## 6. Permissions

Written consent was given by all the participants in the NIPCON study in accordance with the Detroit and Wayne State University School of Medicine Institutional Review Board. The H09-62-94 study was conducted in accordance with the Declaration of Helsinki and approved by the above Institutional Review Boards for studies involving humans. Informed consent was taken from all the subjects involved in this study. Online data for the PD-L1 data sources are given in the body of the manuscript or in the References Section and were obtained during the month of November 2023. The views expressed herein are purely those of the authors and do not necessarily reflect those of the United States Federal Government under whose auspices this study was conducted.

## Figures and Tables

**Table 1.** Comparison of Cancer Type with Demographics and FERAD and NLR Denominators.

Parameters	Age/Gender/Race	FERAD/NLR rb	FERAD/NLR rc	*p* Value
Cancers (n)	Patient Demographics	x ± Standard Dev.	x ± Standard Dev.	
Breast (7)	58.20/1M6F/3B4W	3450 ± 6220 ˆ	31,099 ± 70,229 *	0.07*
Colorectal (4)	58.00/3M1F/3B1W	12,596 ± 21,078	23,177 ± 30,729 ˆ	<0.0006 ˆ
Prostate (17)	58.47/17M0F/10B7W	23,680 ± 85,521	27,996 ± 190,474	NSS
Bladder (3)	64.00/3M0F/0B/3W	23,680 ± 85,521 #	119,125 ± 30,794 #	<0.038 #
Hepatoma (3)	48.67/3M0F/2B1W	228,995 ± 174,102	228,571 ± 384,111	NSS
Melanoma (3)	52.67/3M0F/1B2W	39,581 ± 47,379	478,365 ± 821,187 ‘	<0.013
Pancreas (4)	49.00/1M3F/3B1W	9758 ± 10,859	382,001 ± 30,729	NSS
Lung (7)	50.86/7M0F/4B3W	18,002 ± 33,929	286,243 ± 712,732	NSS

Other Races: Pacific Islander, Columbian, Native American. M—male; F—female, W—white; B—black.

ˆ *p* < 0.0006;

* *p* = 0.07;

# *p* < 0.038;

‘ *p* < 0.013.

**Table 2.** Overview of study parameters.

Parameters	No Adenomas	Insignificant	Significant	Cancers	*p*-Value
Number	759	319	233	42	N/A
Sex Male–Female	577:103 (84.9%)	275:22 (92.6%)	210:9 (95.9%)	39:1 (97.5%)	NSS
Ethnicity AA–Cau	363:293 (55.3%)	161:137 (54%)	123:92 (57.2%)	22:19 (53.7%)	NSS
F/H cancer +:−	263:397 (39.9%)	126:168 (42.9%)	186:131 (58.7%)	17:24 (41.5%)	NSS
Age in Years × ± sd	59.3 ± 12.2	60.9 ± 9.9	62.3 ± 10.6	64.9 ± 11.8	<0.003 *
BMI kg/sqr m	28.6 ± 6.0	29.3 ± 6.3	28.7 ± 6.0	28.3 ± 5.8	NSS
GI Symptoms +:−	428:296 (59.2%)	158:145 (52.2%)	126:93 (57.5%)	27:13 (67.5%)	NSS
Alive–Dead +:−	396:277 (58.8%)	160:141 (53%)	127:92 (58%)	14:27 (34.3%)	NSS cEx
Creatinine mg/dL	1.18 ± 0.93	1.14 ± 0.39	1.59 ± 0.95	1.36 ± 1.02	NSS
Smokers +/−	173:245 (41.4%)	109:121 (47.4%)	82:96 (46.1%)	15:22 (22.4%)	NSS
Ethanol Intake +:−	171:257 (40.0%)	74:114 (39.4%)	57:55 (50.9%)	17:16 (51.5%)	NSS
Flu Vaccination +:−	137:159 (46.3%)	70:100 (41.2%)	63:74 (46%)	8:9 (47.1%)	NSS
Illicit Drug Use	32:193 (14.2%)	21:81 (20.6%)	15:53 (22.1%)	3:18 (14.3%)	NSS
Diabetic	212:394 (35.2%)	110:161 (40.6%)	86:117 (42.4%)	14:27 (34.2%)	NSS
Hepatitis	53:296 (15.2%)	32:132 (19.5%)	14:87 (13.9%)	4:21 (16%)	NSS

N/A—not applicable; NSS—not statistically significant; AA—African American; Cau—Caucasian; F/H—family history;

* —no polyps vs. significant polyps and cancers; x—mean; sd—standard deviation; kg—kilogram; sqr—square; GI—gastrointestinal; cEx—cancer excluded.

**Table 3.** Shows FERAD ratio in premalignant stomach conditions and other cancers of interest.

Parameters	Chronic Atrophy	Intestinal Metaplasia	Family History Cancer	Gastric Cancer
Number	14	25	14	10
Mean FERAD	54,535	10,237	13,384	3966
Standard Dev.	108,018	11,341	31,829	3644

**Table 4.** Demographic and Biological Data in Patients with and without Defined Primaries.

Parameter	Uncertain Primary	Defined Primary	*p* Value
Age × ± sd	57.3 ± 8.9	61.6 ± 10.3	NSS
Gender M–F	12M:0F 100%	28M:3F 90.3%	NSS
Ethnicity B–W	6B:6W 50%	20B:12W 62.5%	NSS
BMI	31.7 ± 7.4	28.3 ± 5.1	NSS
Observation yrs	17.8 ± 5.3	11.7 ± 5.4	<0.004
Diabetes	5 + 7–71.4%	7 + 25–22%	NSS
Hepatitis	2 + 9–18.2%	4 + 18–18.2%	NSS
Smoking Status	6 + 5–64.6%	16 + 13–55.2%	NSS
Ethanol Intake	3 + 7–40%	9 + 16–36%	NSS
N/L ratio	2.85 ± 1.17	3.21 ± 2.61	NSS
Stool p87	0.029 ± 0.042	0.058 ± 0.082	NSS
Effluent p87	0.141 ± 0.282	0.238 ± 0.332	NSS
F/H cancer	4 + 8–33%	4 + 15–21.1%	NSS
Flu Vaccination	4 + 6–40%	5 + 12–29.4%	NSS
GI Symptoms	8 + 4–66.7%	15 + 17–46.9%	NSS

**Table 5.** FERAD ratios in patients with cirrhosis, HCC, or both, versus controls.

Parameter (n)	Mean FERAD Ratio	Standard Dev.	*p* Value
HCC and Cirrhosis (7)	3930	2580	<0.022
HCC only (11)	85,709	150,073	0.41
Cirrhosis only (11)	148,418	322,805	0.33
Controls (10)	42,894	85,866	Comparison

n—number; HCC—hepatocellular cancer; combination of FERAD ratio in HCC patients and cirrhosis that frequently co-exist were ~120,000; Dev—deviation.

## Data Availability

The data presented in this study are available upon request from the corresponding author under the Data Transfer Agreement and all the following conditions apply. An application must contain statements to the effect of the following: what data are being requested; what data may be used; how will the data be used; who will access the data; and how the data will be accessed, stored, and safeguarded. The applicant must also address how the data will be disposed of, after the completion of the data review. Suggested Data Availability Statements are available in the VHA directive 1200.12 of 3/9/2009. The VHA Handbook addresses both the use of data for research and the clinical and administrative data repositories for research. It also addresses the development and use of data research repositories.
